# How Financial Beliefs and Behaviors Influence the Financial Health of Individuals Struggling with Opioid Use Disorder

**DOI:** 10.3390/bs14050394

**Published:** 2024-05-09

**Authors:** James R. Langabeer, Francine R. Vega, Marylou Cardenas-Turanzas, A. Sarah Cohen, Karima Lalani, Tiffany Champagne-Langabeer

**Affiliations:** 1 Center for Behavioral Emergency and Addiction Research, The University of Texas Health Science Center at Houston, Houston, TX 77030, USA; james.r.langabeer@uth.tmc.edu (J.R.L.); francine.r.vega@uth.tmc.edu (F.R.V.); maria.cardenasturanzas@uth.tmc.edu (M.C.-T.); audrey.s.cohen@uth.tmc.edu (A.S.C.); 2 School of Public Health, University of Washington, Seattle, WA 98195, USA; klalani@uw.edu

**Keywords:** financial behaviors, opioids, financial anxiety, financial beliefs

## Abstract

The surge in opioid use disorder (OUD) over the past decade escalated opioid overdoses to a leading cause of death in the United States. With adverse effects on cognition, risk-taking, and decision-making, OUD may negatively influence financial well-being. This study examined the financial health of individuals diagnosed with OUD by reviewing financial beliefs and financial behaviors. We evaluated quality of life, perceptions of financial condition during active use and recovery, and total debt. We distributed a 20-item survey to 150 individuals in an outpatient treatment program for OUD in a large metropolitan area, yielding a 56% response rate. The results revealed low overall financial health, with a median debt of USD 12,961 and a quality-of-life score of 72.80, 9.4% lower than the U.S. average (82.10). Most participants (65.75%) reported improved financial health during recovery, while a higher majority (79.45%) worsened during active use. Unemployment affected 42% of respondents, and 9.52% were employed only part-time. Regression analysis highlighted a strong association between lack of full-time employment and a lack of financial advising with total debt. High financial anxiety and active use were associated with lower quality of life. Individuals with OUD may benefit from financial interventions, resources, and counseling to improve their financial health.

## 1. Introduction

Approximately 68% of the 108,000 deaths resulting from drug overdose were linked to opioids, solidifying it as one of the primary causes of death among substance users [1]. In the United States, approximately 7.6 million individuals are currently living with opioid use disorder (OUD), with nearly 9 million reported cases of OUD in 2022 [2,3]. Notably, during the peak of the COVID-19 pandemic, sociodemographic factors and heightened financial stressors contributed to a surge in opioid-related fatalities across the nation [4]. The diagnosis of OUD relies on criteria outlined in the American Psychiatric Association Diagnostic and Statistical Manual, fifth edition [5]. OUD encompasses a persistent craving for and use of opioids, even in the face of adverse social and professional repercussions. The class of drugs known as opioids includes both prescription painkillers (e.g., codeine, oxycodone) and illicit substances (e.g., heroin or fentanyl). These substances are psychoactive drugs that, when used chronically, can change brain function, including structural abnormalities such as widespread cell death and atrophy. Prior studies have confirmed that long-term substance use disorder (SUD) and OUD often correlate with deficits in decision-making, impulsivity, and executive function [6,7]. Such damage results in mild-to-significant cognitive and behavioral deficits that linger long after detoxification [8,9]. Therefore, substances impacting decision-making could also worsen life skills and financial decision-making.

In addition to cognitive consequences, SUD has far-reaching impacts on social structure, families, employment, and overall financial health. These consequences impact individuals, their families, and local and federal governments. SUD has been linked to a reduction in financial resources that results in poverty, including first-time homelessness [10,11]. The financial strain on family members may result from increased direct help for food, clothes, medical care, and housing while caring for persons with SUD [11]. Some persons with drug disorders may not have family they can rely on for assistance, placing budgetary constraints on local communities and the government. One study found that between 30 and 50% of all adults who are homeless also have SUD [12]. To date, most research on individuals with OUD has focused on treatment and recovery outcomes and not the short- and long-term financial impacts of addiction. To close this knowledge gap, more research is needed to better understand the financial decision-making habits, history of financial attitudes or beliefs, knowledge, and environmental factors of individuals at various stages of recovery. 

### Background

Financial health has been described as one’s ability to manage daily expenses and debt, procure food and housing, and build wealth [13]. Financial health can also be described as financial well-being, which is believed to be influenced by education, social class, work status [14], and political and religious beliefs [15]. Financial literacy, however, is considered one of the most determining factors of financial well-being [16]. Prior research has suggested that financial well-being has two primary dimensions: financial beliefs (i.e., perceptions and attitudes) and financial behaviors or actions [17,18,19]. Other researchers have theorized that financial literacy is critical in changing behaviors and beliefs [20]. There have been attempts to classify beliefs about personal finances and money into various typologies, primarily composed of factors related to how individuals feel about debt, spending versus saving, parental upbringing, emotions, and anxiety that money creates, as well as other dimensions [21]. More recently, Klontz and Klontz hypothesized that every person has one of four money types called scripts [22]. These scripts describe what individuals believe to be true about money or finances and are often developed early in childhood, genetically, and usually subconsciously. The four money subtypes include those who are cautious, those who avoid, those who guard, and those who are spenders. These underlying beliefs or scripts drive an individual’s financial behaviors and financial health. 

Financial literacy may also affect financial decision-making behavior and significantly impact building assets [23]. Financial literacy refers to an individual’s financial knowledge, gauged by their grasp of personal financial concepts. This understanding directly impacts their ability to effectively plan, budget, monitor, manage, and control their financial matters [24,25]. Yet, individuals with SUD often have diminished cognition, and elevation of dopamine, which occurs when opioids are used, has been linked to excessive risk-taking due to reward saliency. Cognitive decline and decreased impulse control may lead to impulsive spending and using money to purchase drugs [26]. Therefore, OUD may decrease sensitivity to financial losses since individuals have a high-risk tolerance to known and ambiguous (prospective) risks [20,27,28,29,30,31]. Drawing upon previous research indicating impaired decision-making abilities and high-risk tolerance, alongside financial studies highlighting the influence of financial beliefs and behaviors, this research seeks to establish a foundational understanding of financial health and its determinants among individuals with OUD.

## 2. Materials and Methods

### 2.1. Study Design

This study utilized a cross-sectional analytic survey design to collect data from a convenience sample of individuals within a treatment and recovery program for OUD in the Houston, Texas, metropolitan area. The survey was developed and tested in March 2023 and administered over a four-week period in June 2023. The survey was distributed electronically using Qualtrics© XM software (version 2024, Qualtrics, Provo, UT, USA). This study was approved as a quality improvement program under the institutional research board of the University of Texas Health Science Center at Houston. All data were secured on the affiliated university’s encrypted server and accessed using password-protected computers. 

### 2.2. Setting

The details of the components and processes utilized to implement this program, the Houston Emergency Opioid Engagement System (HEROES Program), have been published previously [32,33]. Eligible participants were diagnosed with OUD and were within varying stages of recovery.

### 2.3. Participants

Participants were enrolled in a treatment program that provides outreach, counseling, medications, and peer support for individuals who have previously overdosed from opioids. All individuals provided consent to participate, and participation was voluntary. No compensation or exchange of services was provided for participation. 

### 2.4. Variables

The questionnaire consisted of 20 items, organized into four main categories—demographics, financial beliefs, financial literacy, and financial behavior—and was designed to be completed in less than 5 min. Previous studies on financial health and substance use were closely examined to help determine initial questions for inclusion. For money beliefs, we relied upon the four money typologies developed in the Klontz Money Script Inventory, a well-established instrument to explore parent and individual money beliefs and behaviors [34]. The survey, which we included as Supplementary Materials, consisted of a mix of dichotomous questions, choice-based questions, and open-ended questions along the four predetermined dimensions shown in Table 1.

The study outcomes were measured by asking each participant about their total debt or financial obligations, measured in U.S. dollars, and their overall quality of life (QOL). We relied on the European Quality of Life (EQ-5D) visual analog scale ranging from 0 (lowest overall health) to 100 (highest quality of life) [35] and utilized the most recently updated U.S. median norm values as a comparison [36]. We used two other measures of financial health, gauging individuals’ perceptions of their financial condition in both periods of relapse and recovery. The first two measures (debt and QOL) were the primary dependent variables for further analyses. 

### 2.5. Data Sources/Measurement

The research team met collectively to discuss the initial draft of questions, refined the questions, and identified the following two dimensions: beliefs (i.e., attitudes and perceptions around money and finances) and financial behaviors (i.e., the actions individuals pursue with money). The survey was pre-tested for usability in March 2023 with 5 subjects, and the data collected from the usability test were excluded from our later analyses. 

### 2.6. Bias

For the open-ended questions, we derived iterative sub-themes from the written responses. Two authors (F.V. and A.S.C.) read and coded responses separately, and then themes were developed using cluster analysis in Excel. These were compared and reconciled to address potential reviewer classification bias.

### 2.7. Study Size

We chose to survey all participants who had enrolled in the program during the previous 12-month period, which was 150 participants.

### 2.8. Statistical Analysis

We calculated measures of central tendency for continuous variables and proportions for ordinal and nominal variables. We reported on the overall response rate to the survey and evaluated the distribution of the study variables. Total debt was not normally distributed and was log-transformed to meet the assumptions required to conduct linear regression. We conducted separate univariate linear regression analyses to determine variables associated with our outcomes: log-transformed total debt and quality of life. In the following step, we conducted multivariable linear regression to find variables independently associated with the outcomes. For all other questions, analyses were two-tailed, a *p*-value < 0.05 was considered significant, and analyses were conducted with Stata I/C version 15, Stata Corp LLC, College Station, TX, USA.

## 3. Results

### 3.1. Participants

Out of 150 participants invited to participate electronically, 88 (58.7%) unique surveys were received. Four questionnaires were excluded from the analyses because of a lack of responses in more than 25% of the survey questions, resulting in 84 respondents in the final analysis (56% response rate).

### 3.2. Descriptive Data

We used complete case analysis and excluded missing data from the analyses. The proportion of female participants was higher than that of males (52.38% vs. 45.24%), and two participants (2.38%) identified as non-binary or preferred not to say. The respondents spanned several age ranges, with the largest group of responses from individuals between the ages of 35 and 44 (48.19%). Eleven respondents identified as college graduates or had a postgraduate education (13.10%), while 30.95% had a high school diploma as their highest level of education. Nearly 42% (n = 35) were unemployed during the study, and another 9.58% (n = 8) were employed only in a part-time position. More than half (53.01%) had been diagnosed with SUD for 10 years or more (n = 44). Table 2 presents a summary of the demographic characteristics and indicators of financial health.

### 3.3. Outcomes

The median total debt reported was USD 12,961 (1600–29,000). This was highly skewed, however, by a low response rate on this question (64.3%, n = 54), requiring log transformation for further regression analyses. The mean QOL was 72.8 (sd, 20.28). As a point of comparison, the U.S. population norm for the EQ-5D QOL scale is 80.4 [36]. This suggests that individuals with OUD in this sample had 7.6 points (or 9.4%) lower quality of life than the U.S. population across similar age ranges. 

Nearly 66% (48) felt their financial health improved during periods of recovery, and 79.45% felt their finances worsened significantly during periods of active substance use.

### 3.4. Main Results

#### 3.4.1. Financial Beliefs and Behaviors

Over 63% (53) of respondents reported they were not adequately paid for their skills. Regarding perceptions of importance, the majority (72.6%) stated that insufficient income was more important than reducing uncontrolled expenses (27.40%). Of the four money typologies measured for money belief (variable self_belief), the largest proportion (34.25%) indicated they were vigilant with their money, saying they prefer to save rather than spend. The second largest category was those who preferred to spend money today (28.77%), and an equal proportion (28.77%) said they avoided the concept of money altogether or perceived money as evil (avoiders). Only 8.22% said they hoarded money. The distribution was similar to what respondents felt their parents’ beliefs about money were (45.21% vigilant, 24.66% spenders, 23.29% avoiders, and 6.85% hoarders). There was no significant correlation between money beliefs between the individual and their parents. 

A majority (61.64%) felt financial anxiety or reported being stuck with their financial situation, and 72.60% felt their bigger problem was not having enough income versus being unable to control their spending (27.40%). Respondents were asked to describe what would help them improve their financial situation in an open-ended question. Using the coding methods described earlier, results suggest that the most common response was a need for better work or disability income (23, 32%), followed by the use of a budget or better spending behaviors (16, 22%), staying sober or healthy (13, 18%), and receiving help from others with their money (12, 16%). 

The majority (48, 65.75%) reported not using a written budget for managing their spending or expenses. Only 21.92% (16 of 73) had previously utilized a financial planner, advisor, or coach to help with their financial situation. When responding to the hypothetical question of what they would do with an unexpected USD 1000 windfall income, 42% said they would pay existing bills, 23% would save it, and 19% would give the funds to friends or family.

#### 3.4.2. Total Debt

We observed four variables strongly associated with total debt. Based on these analyses, we found that the average debt of participants with a part-time job was 83% less than that of participants who were not employed. The average debt of unemployed respondents was USD 14,553, compared to USD 2474 for those with part-time employment (*p* < 0.05). Debt also varied significantly by use of a financial advisor as well. The average debt of participants who had previously used a financial advisor was approximately 65% less than those without a prior financial advisor or advice from someone with financial training (USD 14,913 vs. %5220, *p* ≤ 0.003). 

Regarding financial behavior and regardless of how the financial situation changed over periods of substance use, we found that the low number of observations in this category made this analysis unreliable. The average rate of debt for the seven participants who reported a worsened situation decreased by 47% compared to those who reported that their situation was the same. The average debt of participants who reported their financial situation stayed the same during periods of substance use was USD 38,217.55, in contrast with those who reported their financial situation worsened, whose debt was only USD 20,255.30 (*p* < 0.05). Table 3 represents the separated univariate linear regression analyses of our two primary outcome variables.

#### 3.4.3. Total Debt Regression Results

We then entered the significant variables into a multivariate regression and found three characteristics persisted as inversely associated with total debt: employment status (having a part-time job, *p* = 0.01); having had a prior financial advisor or coach (*p* = 0.005); and financial health worsening during periods of substance use (*p* = 0.04). Table 4 presents the regression findings.

#### 3.4.4. Quality of Life Regression Results

We found seven characteristics associated with quality of life: employment status, adequate pay if employed (belief), feeling stuck when it comes to finances (belief), uncontrolled purchases (beliefs), money vigilance (behavior), having a written budget (behavior), and perception of improvement in financial health during recovery (health). A two-way ANOVA examined interactions of covariates with total debt (log). No significant interaction was found between gender and employment or gender and financial situation during periods of substance abuse. The covariates examined for interactions using two-way ANOVA for the outcome of quality of life resulted in no significant interactions. Three of these variables persisted in multivariate analyses (age 25 to 35 was positively associated with QOL (*p* = 0.004), financial anxiety was negatively associated with quality (*p* = 0.002), and improved financial health during recovery was also positively associated with QOL). Table 5 presents the multivariate results.

## 4. Discussion

### 4.1. Key Results

Prior research has established that OUD impacts social and family systems, as well as individual behavior and decision-making. Theories of social determinants of health suggest that recovery requires housing, food, and transportation stability to improve overall health. Yet, few studies have focused specifically on the financial implications of OUD. In this study, we examined financial beliefs, behaviors, and outcomes to provide evidence of an association between individuals with OUD and their overall financial health. Specifically, our findings support the idea that beliefs and behaviors do have significant associations with the financial health of individuals in addiction treatment.

Interestingly, our sample had a 9.4% lower overall QOL than the median quality of life for the U.S. population for similar age groups. Quality of life is an important indicator of an individual’s overall well-being. Lower self-assessments could be related to the impact of opioid use disorders in general or a reflection of their current financial position. In addition, after controls and in the presence of multiple variables, total debt was significantly higher for those with part-time employment, those who had never used a financial advisor or coach, and those who believed their situation worsened when they were in active use or relapse periods. Quality of life varied by age and was significantly lower for those aged 25–34. Feelings of financial anxiety or being stuck were associated with lower quality of life. These findings suggest that policies and practices that offer ancillary resources, such as an advisor or financial coach, and assistance with employment could help individuals with budgeting, life skills, and reducing debt load for people in recovery. Similarly, helping individuals focus on reducing anxiety over finances and staying in recovery can help improve overall quality of life. 

Although this study was unique in its focus on individuals with OUD, our findings agree with research in other treatment centers. One study showed that over 50% of individuals in one addiction treatment center did not have access to financial accounts or services from traditional financial institutions, and low levels of financial literacy compounded their challenges in recovery [37]. Another study found that financial anxiety and concerns about managing financial commitments while being in treatment were the dominant concerns of both patients and their parents [38]. Financial anxiety is important for additional research. Social determinants of health (SDOH) are well known to impact individuals with a history of OUD. Challenges these individuals face include loss of employment, homelessness, and increased likelihood of incarceration, all of which promote anxiety. Loss of a job or employer benefit due to the disease leads to worsened financial anxiety and potential quality of life. During the recovery phase, individuals with OUD also report similar challenges, specifically difficulty finding adequate employment and challenges with housing stability, as well as stigma and bias [39].

Working on improving an individual’s deeply rooted beliefs about money is also important. Although the highest proportion of this sample reported they were vigilant about saving and spending, these beliefs impact our financial behaviors and are potential factors related to lower financial health. Debt is an important area of concern and anxiety for many people in recovery. Addictions are known to create excessive spending during periods of active use, creating financial burdens [40]. Although, in this study, we found that the average debt was potentially lower than state averages compared to the general population (~USD 13,000), we attribute this largely to the fact that most individuals in OUD treatment are less likely to own a home or have large sums of debt from larger purchases such as cars [41]. Municipal recovery centers and addiction treatment centers could also offer additional resources to patients, such as financial literacy campaigns for recovery with upskilling resources for those trying to find work [20]. How to deploy financial literacy campaigns effectively for people in recovery should be further explored.

Together, our analyses provide limited evidence that certain financial beliefs and behaviors do influence financial health in this sample of people with opioid use disorder. Further research could include a different analysis of finances pre- and post-recovery and obtain subscale scores around financial beliefs, behavior, literacy, and other potential dimensions. Additional questions could better examine SDOH in relation to ethnic groups with deeper questions about financial behavior. 

### 4.2. Limitations

There are several limitations to this study. First, it is a small sample size and represents individuals with opioid use disorder, which may not represent other substance use disorders. Second, this survey design has inherent limitations, particularly the risk of social desirability bias in reporting financial improvement while in recovery. Third, this study utilized a convenience sample, and the format allowed for open-ended responses to certain questions. 

### 4.3. Interpretation and Conclusions

In this study examining the financial behaviors and beliefs of individuals living with OUD, we found that financial resources are a source of anxiety for individuals. For many people who enter treatment, their recovery does not include a focus on improvement in personal finance, which is problematic for both society and the individual. Our findings support the need to provide financial resources (financial advising or counseling) and focus on financial recovery capital and skill-building resources for individuals in treatment. Improving financial knowledge and literacy, as well as changing money beliefs and behaviors, could help to improve the financial conditions of people in recovery and create a meaningful plan for improving overall financial health.

### 4.4. Generalizability

This study used a convenience sample of participants in one drug treatment program. Due to a low number of responses, the small sample size resulted in large confidence intervals, which suggests that these results may not fully represent the population. Caution should be taken with interpretation and translation to the general population.

## Figures and Tables

**Table 1:** Dimensions and variables of survey distributed to study participants.

Dimension	Question Focus/Variable Name	# of Questions
Demographics	Gender (sex)	6
	Educational level (education_years)	
	Employment status (employment)	
	Age ranges (age)	
	Co-occurring mental health (mental_health)	
	Length of diagnosis (diagnosis_years)	
Financial Beliefs	Are you adequately paid for your skills? (pay)	7
	Do you feel anxious or stuck with finances? (fin_anxiety)	
	What beliefs did your parents have towards money? (parent_belief)	
	What is your attitude towards money? (self_belief)	
	What money challenges do you identify best with? (challenge)	
	What would it take to improve your financial situation? (fin_needs)	
	Do you feel your finances are stable? (stability)	
Financial Behaviors	Do you currently use a written budget? (budget)	3
	Have you ever used a financial planner? (advisor)	
	What would you do with an additional USD 1000 windfall income? (behavior_windfall)	
Financial Health	What is your est. of total debt or financial obligations? (debt)	4
	Current perceived quality of life (QOL)	
	How has your financial condition changed during active substance use? (activeuse_health)	
	How has your financial condition changed since recovery? (recovery_health)	

**Table 2:** Sociodemographic and financial health characteristics of study sample.

Characteristic	N (%)
Total	84 (100)
Age group	
Less than 24 years	5 (5.95)
25 to 34	20 (23.81)
35 to 44	40 (47.62)
45 or older	19 (22.62)
Gender (sex)	
Male	38 (45.24)
Female	44 (52.38)
Non-binary or unknown	2 (2.38)
Education level	
Less than high school degree	7 (8.33)
HS graduate (HS diploma, GED or equivalent)	26 (30.95)
Some college but no degree	37 (44.05)
Degree obtained (associate, bachelor’s, or graduate degree)	14 (16.67)
Employment status	
Full time (40 h)	41 (48.81)
Part time	8 (9.52)
Not employed	35 (41.67)
Diagnosis of co-occurring mental health condition	
Yes	37 (44.05)
No	47 (55.95)
SUD length of diagnosis	
<5 years	14 (16.87)
5 years but <10	22 (26.51)
10 years or more	47 (56.63)
Financial health outcomes	
Total debt, mean (sd)	USD 12,961 (1600–29,000)
Quality of life, QOL, median (IQR)	72.80 (20.28)

Note: N may not add up to 84 due to missing responses on certain questions.

**Table 3:** Univariate linear regression of demographic characteristics, financial beliefs, and behaviors associated with total debt and QOL.

Characteristics	Study Outcome: Total Debt *			Study Outcome: QOL Score		
Socio-Demographic	Coefficient	Beta	*p*-Value	Coefficient	Beta	*p*-Value
Age group						
Less than 24 years	0.18	0.21	0.88	−4.05	−0.05	0.66
25 to 34	−0.11	−0.30	0.86	−12.45	−0.26	0.06
35 to 44	0.67	0.21	0.23	−5.05	−0.13	0.38
45 or older	9.01 **	NA		78.05 **	NA	
Gender ***						
Male	0.36	0.11	0.42	−6.75	−0.17	
Female	9.19 **			9.19 **	NA	0.14
Education						
Less than high school degree	8.01 **	NA		73.29 **	NA	
HS graduate (HS diploma, GED or equivalent)	1.18	0.35	0.48	−9.13	−0.21	0.28
Some college but no degree	1.59	0.50	0.34	1.71	0.04	0.83
Associate degree, bachelor’s degree, or graduate degree	0.63	0.13	0.72	8.86	0.16	0.33
Employment						
Full time (40 h)	−0.22	−0.07	0.63	12.61	0.31	**0.007**
Part time	−1.83	−0.33	**0.02**	8.61	0.13	0.27
Not employed	9.59 **	NA		65.89 **	NA	
Diagnosis of mental health condition						
Yes	0.0004	0.0001	0.99	−0.85	−0.02	0.85
No	9.31 **	NA		73.17 **	NA	
Diagnosis duration						
Less than 5 years	0.22	0.05	0.73	−3.15	−0.06	0.62
5 years to less than 10 years	−0.12	−0.03	0.81	−2.30	−0.05	0.67
More than 10 years	9.31 **	NA		73.94 **	NA	
*Financial Beliefs*						
Adequately paid for your skills						
Yes	9.11 **	NA		79.43 **	NA	
No	0.30	0.09	0.53	−10.40	−0.25	0.02
How would you describe your parents’/guardians’ attitudes with regard to money?						
Hoarders	−1.04	−0.15	0.30	17.08	0.22	0.08
Vigilance	−1.43	−0.44	**0.008**	15.19	0.38	**0.009**
Spenders	−0.50	−0.13	0.41	19.94	0.43	**0.003**
Avoiders	10.14 **	NA	NA	60.12 **	NA	NA
How would you describe yourself with regard to money?						
Hoarders	−0.30	−0.06	0.72	3.86	0.05	
Vigilance	−0.84	−0.24	0.16	21.91	0.52	0.65
Spenders	−0.84	−0.25	0.15	8.48	0.19	**≤0.001**
Avoiders	9.88 **	NA	NA	62.81 **	NA	0.14
What is more important to you?						
Not having enough income	9.44 **	NA		69.40 **	NA	
Uncontrolled purchases	−0.54	−0.15	0.30	13.40	0.30	**0.01**
Do you feel stuck when it comes to your finances (like you do not know what to do to turn things around)?						
Yes	0.80	0.24	0.08	−19.24	−0.47	**≤0.001**
No	8.79 **	NA		84.93 **	NA	
*Financial Behaviors*						
Do you have a written budget for your income and expenses?						
Yes	9.08 **	NA		79.74 **	NA	
No	0.32	0.09	0.51	−9.99	−0.24	0.04
What would you do if you had an additional USD 1000.00 today?						
Pay off debt	9.17 **	NA		71.72 **	NA	
Spend it on something good	−0.65	−0.06	0.70	21.03	0.24	0.05
Use it for …	0.28	0.09	0.55	−0.09	−0.01	0.99
Give it away	-	-	-	18.28	0.11	0.37
Have you ever hired or talked to someone about your finances, such as a financial planner, financial coach, or adviser?						
Yes	−1.65	−0.40	**0.003**	5.84	0.12	
No	9.61 **	NA		71.79 **	NA	0.31
*Financial Health*						
How has your financial situation changed during periods of substance use?						
Improved	−0.80	−0.14	0.41	15.73	0.23	0.13
Worsened	−1.47	−0.37	**0.04**	11.88	0.24	0.12
Stayed the same	10.55 **	NA		62.13 **	NA	

* This variable was log-transformed to achieve a normal distribution; ** linear regression constant; *** excluded one case of unknown gender; ***p*-value significant**, bold; QOL, quality of life score; NA, not available.

**Table 4:** Multivariate linear regression analysis of predictors associated with total debt.

Characteristic	Coefficient	Beta	*p*-Value
Employment			
Full time (40 h)			
Part time	−0.02	−0.01	0.96
Not employed (reference)	−1.77	−0.32	**0.01**
Advisor			
Yes			
No (reference)	−1.46	−0.35	**0.005**
Active_finance			
Improved	−0.80	−0.14	0.36
Worsened	−1.29	−0.32	**0.04**
Stayed the same (reference)			

Note: the variance explained by the model was 25% (adjusted R2 = 0.25). Link test indicated _hat *p*-value = 0.06 and _hat2 *p*-value = 0.13; ***p*-value significant**, bold.

**Table 5:** Multivariate linear regression analysis of predictors associated with qualify of life.

Characteristic (n = 73)	Coefficient	Beta	*p*-Value
Age			
Less than 24 years	−9.33	−0.11	0.34
25 to 34	−17.47	−0.38	**0.004**
35 to 44	−2.25	−0.06	0.66
45 or older (reference)			
Employment			
Full time (40 h)	−8.58	−0.22	0.10
Part time	−5.0	−0.08	0.51
Not employed (reference)			
Pay			
Yes (reference)			
No	−2.25	−0.05	0.64
Budget			
Yes (reference)			
No	0.05	0.01	0.99
Challenge			
Not having enough income (reference)			
Uncontrolled purchases	8.14	0.18	0.09
Fin_Anxiety			
Yes			
No (reference)	−15.04	−0.37	**0.002**
Recovery_finance			
Improved			
Worsened	14.40	0.34	**0.006**
Stayed the same (reference)	−7.82	−0.13	0.29
Behavior_windfall			
Pay off debt (reference)			
Spend it on something good	10.91	0.13	0.25
Use it for …	−6.22	−0.15	0.16
Give it away	3.43	0.02	0.85

Note: the variance explained by the model was 38% (adjusted R2 = 0.38). Link test indicated _hat *p*-value = 0.003 and _hat2 *p*-value = 0.03.; ***p*-value significant,** bold.

## Data Availability

Data from this study may be obtained by reasonable request by contacting the corresponding author.

## Supplementary Materials

The following supporting information can be downloaded at: https://www.mdpi.com/article/10.3390/bs14050394/s1. Survey: Financial Practices of Individuals in OUD Treatment.
