# Supplementary material for: How Financial Beliefs and Behaviors Influence the Financial Health of Individuals Struggling with Opioid Use Disorder
Source: Behav Sci (Basel). 2024 May 9;14(5):394. doi: 10.3390/bs14050394 (PMC11117791; doi:10.3390/bs14050394)
Supplement: Supplementary file 1 [file behavsci-14-00394-s001.zip › behavsci-2924525-supplementary.pdf]

Supplementary Materials
Survey: Financial Practices of Individuals in OUD Treatment

Substance use disorders (SUD) can often create financial and budget challenges for people. Please take 5-10 minutes to complete this brief survey to help us better understand your current financial condition and any successes or challenges you may face. These results are completely anonymous (no names or identifiers are collected). Please complete all questions.

Thank you,
UTHealth

Q1. What is your sex?

- ○ Male
- ○ Female
- ○ Non-binary or prefer not to say

Q2. What is the highest level of school you have completed or the highest degree you have received?

- ○ Less than high school degree
- ○ High school graduate (high school diploma or equivalent including GED)
- ○ Some college but no degree
- ○ Associate degree in college (2-year)
- ○ Bachelor's degree in college (4-year)
- ○ Graduate degree

Q3. Are you currently employed?

- ○ Full-time (40 hours/week)
- ○ Part-time (tell us # of weekly hours below)
- ○ Not-employed

Q4. Do you feel you are adequately paid for your skills?

- ○ Yes
- ○ No

Q5. Has a professional, such as your counselor or physician, diagnose you as having a mental health condition (such as depression, bipolar disorder, anxiety)?

- ○ Yes
- ○ No

Q6. How long have you been diagnosed with or first recognized substance use disorder?

- ○ Less than 6 months ago
- ○ 6 months to 1 year ago
- ○ 1 - 2 years ago
- ○ 2 - 3 years ago
- ○ 3 - 4 years ago
- ○ More than 5 years but less than 10 years
- ○ More than 10 years ago
- ○ Other ____

Q7. How old are you?

- ○ Under 18 years
- ○ 18 - 24 years
- ○ 25 - 34 years
- ○ 35 - 44 years
- ○ 45 - 54 years
- ○ 55 - 64 years
- ○ 65 - 74 years
- ○ 75 years or older

Q8. On a scale of 1 to 100 (with 100 being the best health state possible), how would you rate your quality of life?

0 10 20 30 40 50 60 70 80 90 100

Quality of life	

The following questions examine your history on financial beliefs and behaviors

Q9. What money challenges do you best identify with?

- ◯ Not having enough income
- ◯ Uncontrolled purchases

Q10. How would you describe YOUR PARENTS/guardians (those who raised you) with regards to money?

- ◯ The more they saved, the better they felt they were, they rarely spend money
- ◯ They preferred to save rather than spend and had money in savings, though they would spend money occasionally
- ◯ They preferred to spend money, they worked hard for their money and believed it was better to use it now
- ◯ Money was the root of all problems in the world

Q11. How would you describe YOURSELF with regards to money?

- ◯ I hold on to money, the more I have saved the better off I am, I rarely spend money
- ◯ I have money in savings, I prefer to save rather than spend but will spend money occasionally
- ◯ I prefer to spend my money, I work hard for my money better to use it now
- ◯ Money is the root of all problems in the world

Q12. Do you have a written budget for your income and expenses?

- ◯ Yes
- ◯ No

Q13. Do you feel "stuck" when it comes to your finances (like you don't know what to do to turn things around)?

- ○ Yes
- ○ No

Q14. Have you ever hired, or talked to someone about your finances such as a financial planner, financial coach, or adviser?

- ○ Yes
- ○ No

Q15. How has your financial situation changed during periods of substance use?

- ○ Improved
- ○ Worsened
- ○ Stayed the same

Q16. Has your financial situation changed since you started recovery?

- ○ Improved
- ○ Worsened
- ○ Stayed the same

Q17. What specifically do you think it will take to improve your situation?

____

Q18. How much total debt in $ (including credit cards, back rent, car, house, student loans, title loans, or loans to family) do you owe today?

____

Q19. Do you feel your finances are "stable" or are they constantly in flux?

- ○ Yes
- ○ No

Q20. What would you do if you had an additional $1,000 today?

- ○ Spend it on something good
- ○ Pay off debt
- ○ Give it away
- ○ Use it for ... ____

End of Survey

We thank you for your time spent taking this survey.
